# System for delivering microwave ablation to subcutaneous tumors in small-animals under high-field MRI thermometry guidance

**DOI:** 10.1080/02656736.2022.2061727

**Published:** 2022

**Authors:** Jan Sebek, Tej B. Shrestha, Matthew T. Basel, Faraz Chamani, Nooshin Zeinali, Ivina Mali, Macy Payne, Sarah A. Timmerman, Pegah Faridi, Marla Pyle, Martin O’Halloran, M. Conall Dennedy, Stefan H. Bossmann, Punit Prakash

**Affiliations:** a Department of Electrical and Computer Engineering, Kansas State University, Manhattan, KS, USA; b Department of Circuit Theory, Czech Technical University in Prague, Prague, Czech Republic; c Department of Anatomy and Physiology, Kansas State University, Manhattan, KS, USA; d Department of Chemistry, Kansas State University, Manhattan, KS, USA; e College of Medicine, Nursing and Health Sciences, National University of Ireland Galway, Galway, Republic of Ireland; f Department of Cancer Biology, University of Kansas Medical Center, Kansas City, KS, USA

**Keywords:** Small-animal ablation system, magnetic resonance imaging, microwave ablation, MRI thermometry

## Abstract

**Purpose:**

Bio-effects following thermal treatments are a function of the achieved temperature profile in tissue, which can be estimated across tumor volumes with real-time MRI thermometry (MRIT). Here, we report on expansion of a previously developed small-animal microwave hyperthermia system integrated with MRIT for delivering thermal ablation to subcutaneously implanted tumors in mice.

**Methods:**

Computational models were employed to assess suitability of the 2.45 GHz microwave applicators for delivering ablation to subcutaneous tumor targets in mice. Phantoms and *ex-vivo* tissues were heated to temperatures in the range 47–67 °C with custom-made microwave applicators for validating MRIT with the proton resonance frequency shift method against fiberoptic thermometry. HAC15 tumors implanted in nude mice (n = 6) were ablated in vivo and monitored with MRIT in multiple planes. One day post ablation, animals were euthanized, and excised tumors were processed for viability assessment.

**Results:**

Average absolute error between temperatures from fiberoptic sensors and MRIT was 0.6 °C across all *ex-vivo* ablations. During *in-vivo* experiments, tumors with volumes ranging between 5.4–35.9 mm^3^ (mean 14.2 mm^3^) were ablated (duration: 103–150 s) to achieve 55 °C at the tumor boundary. Thermal doses ≥240 CEM43 were achieved across 90.7–98.0% of tumor volumes for four cases. Ablations were incomplete for remaining cases, attributed to motion-affected thermometry. Thermal dose-based ablative tumor coverage agreed with viability assessment of excised tumors.

**Conclusions:**

We have developed a system for delivering microwave ablation to subcutaneous tumors in small animals under MRIT guidance and demonstrated its performance *in*-*vivo*.

## Introduction

Small animal tumor models are common and widely accepted as important tools for the evaluation of various therapeutic approaches [1–4]. Thermal ablation and mild hyperthermia are under investigation for diverse applications including tissue destruction and its combination with other therapeutics such as localized drug delivery or radiation [5–10]. The biological effects of thermal therapies are a function of the temporally and spatially dependent temperature distribution in tissue [11]. A challenge with clinical delivery of thermal ablation is the variability in thermal profiles and thus overall ablation zone profiles across subjects due to factors such as tissue heterogeneity, large blood vessels, or differences in tissue physical properties – such as electrical conductivity and blood perfusion rates – across subjects and tissue type [12–14]. In the case of small-animal thermal therapy, geometric effects are another source of variability (e.g., position of the target tumor relative to the heating applicator), which may also considerably impact observed thermal profiles due to the small size of target volumes relative to the applicator. In a prior mild hyperthermia study, the rate of heating in tissue varied by ~2–3x across hyperthermia exposures in animals where the same applied energy settings were used [15]. Thus, it can be helpful to track thermal profiles during heating to verify desired thermal doses are delivered to targeted tissue and thereby contribute to analysis and interpretation of experimental outcomes. Additionally, monitoring of temperature profiles can contribute to limiting off target heating and limiting toxicities, which may be especially important for survival studies. While invasive temperature sensors, such as thermocouples and fiberoptic sensors provide temperature information at specific points, MRI provides a means for assessing volumetric thermal profiles, albeit at the cost of added system complexity.

While there are several studies employing microwave ablation (MWA) in small animal tumor targets, there are few reports describing technical details about the instrumentation used for delivering heating, which would be important for characterizing the power absorption and thermal profiles. Since MWA devices designed for ablation of human tumors typically yield >15 mm long ablation zones [16], due to the relatively long wavelength at the commonly used frequencies of 915 MHz and 2.45 GHz, these devices are ill suited to ablation of tumors in small animals that can be as small as 5 mm. Nevertheless, several studies have employed clinical MWA devices (~1.5–2 mm in diameter) in small-animal studies with the devices inserted into subcutaneous tumors *via* a percutaneous approach [17–22], or into tumor/normal tissue under open surgery [23–25]. For tumor targets ~5 mm in diameter, insertion of *a* ~1.5–2 mm device results in a substantial fraction of the tumor volume affected by mechanical trauma. A limitation of most studies is the lack of information regarding the thermal profiles in tissues, with few studies reporting temperature data measured or anticipated within tumors and surrounding tissue. Knowledge of thermal dose delivered to tumor and adjacent tissue may be especially important for studies investigating combination of ablation with other therapeutic modalities [26].

Some studies have described the design and characterization of thermal ablation devices specific for use in experimental studies in small animals. Yoon *et al* reported on a broadband planar coaxial microwave applicator [27] which was used to comparatively assess the impact of microwave frequency on ablation profiles in xenografted mice. Skin surface thermal profiles during ablation recorded with an infrared camera revealed more concentrated heating at 18 GHz compared to 2 GHz. Moon *et al* designed a 1.2 mm diameter dual slot antenna operating at 8 GHz with the goal of achieving spherical ablation zones for treatment of ~1 cm diameter tumors in mice [28], with the hypothesis that the relatively high frequency would afford a shorter active antenna length and more concentrated power absorption profile. The device was found to be suitable for ablating ~10–15 mm tumors in rodents and was subsequently used to create ~11 mm ablation zones in normal rat liver. Both of these devices require insertion into the targeted tissue region. Devices designed for ablation of small animal tumors using other energy modalities have been reported, including radiofrequency current [29] and ultrasound [30].

Our group has previously developed a system for MRI thermometry (MRIT) guided delivery of microwave-induced hyperthermia to experimental small animals [15,31]. The system incorporated a directional microwave antenna integrated within a high-field 14.1 T small-animal MRI scanner. Accuracy of temperature measurements as estimated by MRIT was evaluated for mild heating up to 45 °C [31,32] demonstrating the ability of the applicator to deliver mild hyperthermia (40–45 °C) to small animal tumor volumes. The system has also been applied for delivering mild hyperthermia to experimental animals outside the MRI environment and integrated with intra-vital microscopy for monitoring thermally triggered drug release from temperature sensitive liposomes [33]. The suitability of this system for delivering MRI-guided ablative heating to implanted tumors has not previously been assessed. While we anticipated increasing applied power levels would yield ablative temperatures, an important part of this study was characterizing the extent of the ablation zone within targeted and non-targeted tissue regions to assess suitability for *in vivo* small-animal investigations. Similarly, MR thermometry with the proposed sequence was not previously validated for ablative heating at high-fields; since data acquired with this system will be used to quantitatively assess thermal profiles in future studies, we believe it is important to benchmark thermometry accuracy, even in the event that the accuracy is similar to that at lower temperature ranges.

The objectives of this work were 1) to expand the previously developed system for MRIT guided hyperthermia for the thermal ablation studies in small animals and characterize thermal profiles, 2) to validate the accuracy of volumetric temperature readings from MRIT at 14.1 T by comparison with temperature data from fiber optic sensors during heating in phantom and *ex vivo* tissue and 3) to demonstrate use of the system for delivering ablation to subcutaneous HAC15 tumors in nude mice *in vivo*.

## Methods

### System overview

**Figure 1.**
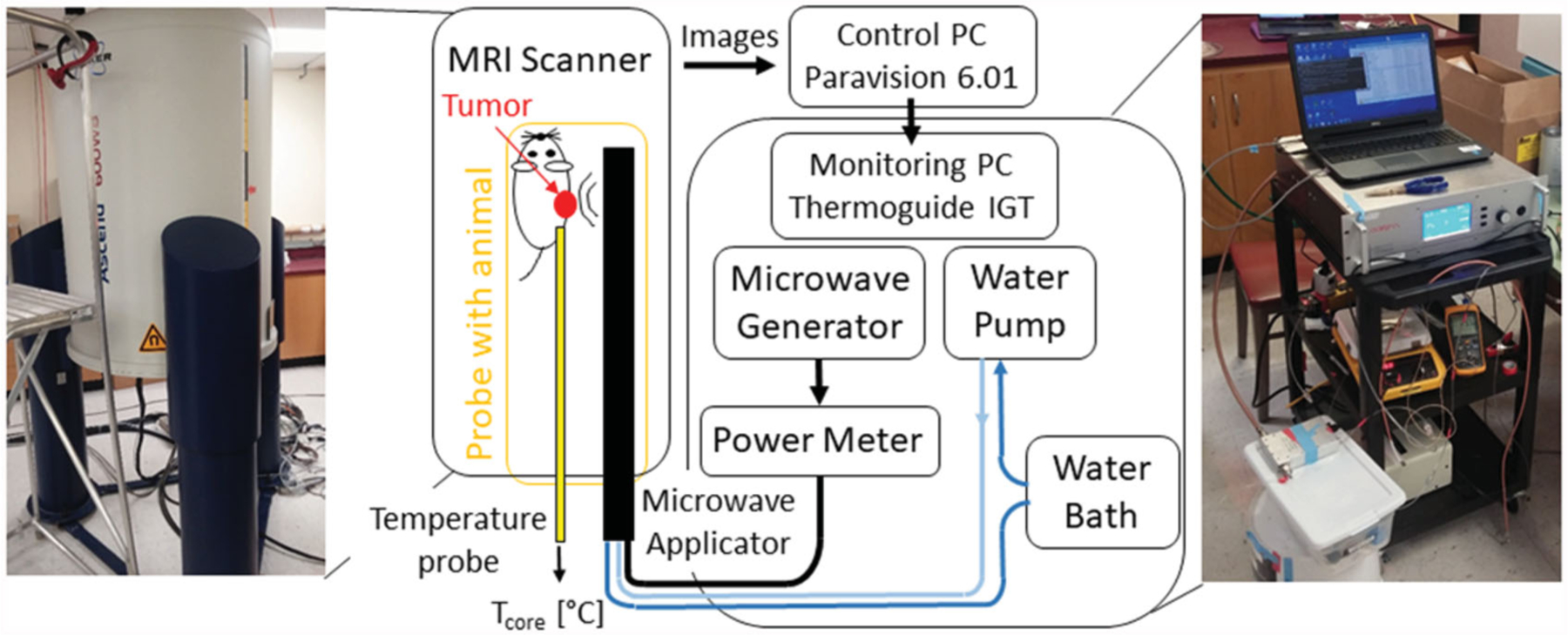
System setup for *ex vivo* as well as *in vivo* ablations with MRIT temperature monitoring.

The small-animal ablation system is comprised of a custom 2.45 GHz microwave ablation platform integrated with a high-field small-animal MRI scanner. The overall schematic of the system is shown in Figure 1. The high-field MRI scanner is a 14.1 T vertical scanner (Bruker Avance III, MA, USA) operating at 600 MHz, and is suitable for high-field imaging of samples that can fit within 2–3 cm diameter imaging coils. In the present study, MR images were acquired with a 30 mm diameter solenoidal coil. The scanner is controlled *via* Paravision 6.01 software. Specific details of imaging sequences, which were used to acquire anatomic images of *ex vivo* and *in vivo* samples are provided in sections ‘System integration and evaluation in tissue phantoms and *ex vivo* tissue’ and ‘*In Vivo* heating procedure’, respectively.

During microwave heating exposure, acquired magnitude and phase images are transmitted *via* ethernet cable to a monitoring and control computer with Thermoguide^tm^ software (Image Guided Therapy, France), where phase images are used to estimate transient temperature profiles with the proton resonance frequency shift (PRFS) technique [34,35]. Imaging sequence details are provided in section ‘System integration and evaluation in tissue phantoms and *ex vivo* tissue’. Since the PRFS technique provides estimates of relative change in temperature, rather than absolute temperature, a fiber-optic temperature measurement probe was utilized to measure the core temperature of the small animal prior to heating, and thus serve as the baseline temperature.

The microwave heating platform consists of a solid-state microwave generator (GMS 200 W SAIREM, France), MRI compatible directional microwave applicator, and a power meter (7022 Statistical, Bird, Solon, OH, USA) for monitoring transmitted and reflected power. A 4 ft long RG393 coaxial cable, with approximate attenuation ~0.12 dB/ft at 2.45 GHz, was used to connect the generator to the power meter, and 6 ft long RG400 coaxial cable, with approximate attenuation ~0.22 dB/ft at 2.45 GHz (Pasternack RF), was used to connect the power meter to the applicator. Details of utilized MWA applicator are provided in the following section. A peristaltic pump (Masterflex L/S 7015–20) was used to circulate water through the applicator from a temperature-controlled water reservoir.

### Small-animal microwave thermal therapy applicator

**Figure 2.**
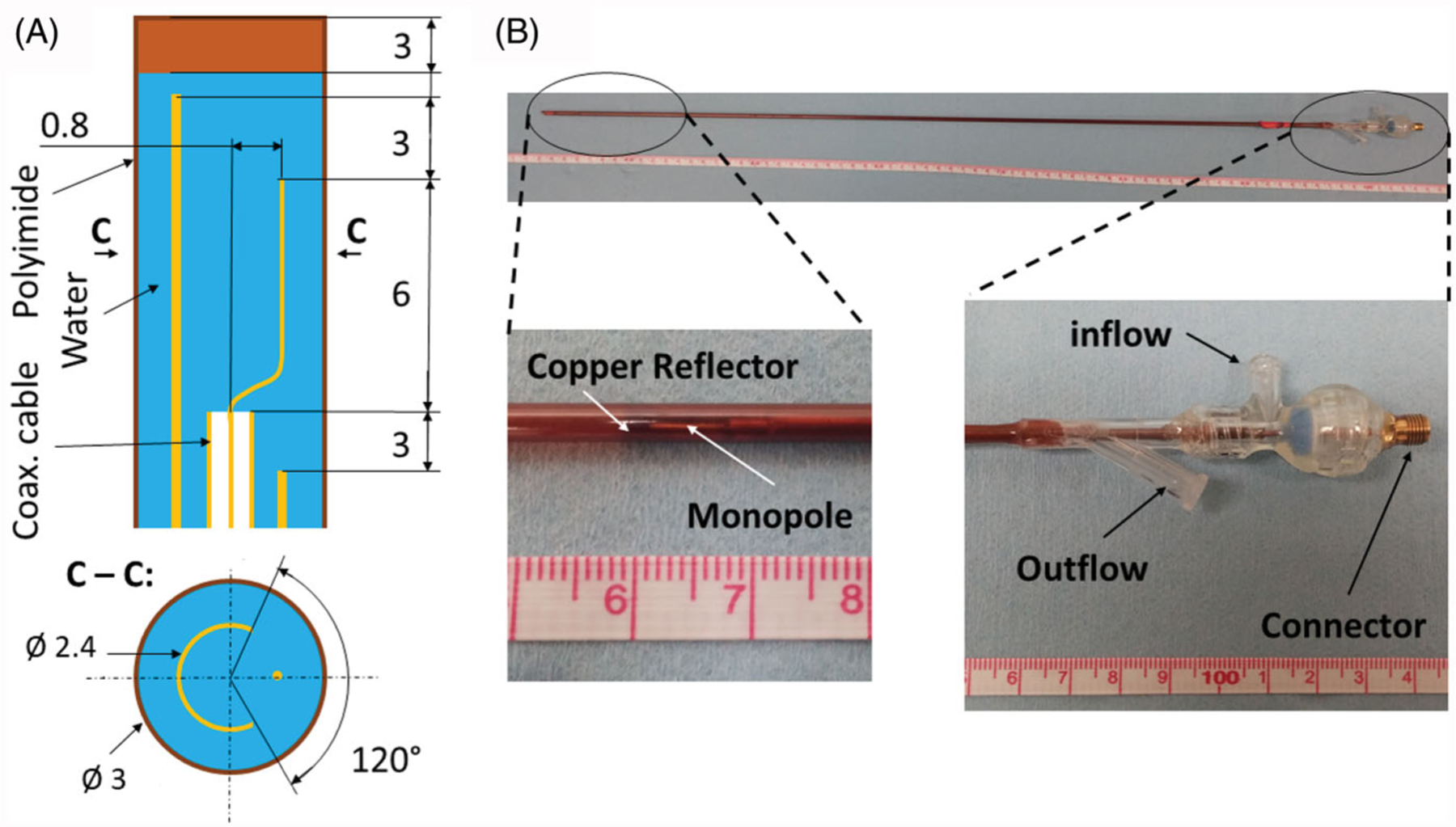
Custom made directional MRI-compatible applicator. (A) geometry of radiating tip. (B) Photo of applicator together with close-up view of the distal radiating tip of the applicator and proximal part with electrical connection and water inflow and outflow lines.

We investigated the feasibility of delivering microwave ablation to subcutaneously implanted experimental tumors in small-animals using a water-cooled microwave applicator previously developed for mild-hyperthermia applications [15, 31]. The applicator was designed to radiate 2.45 GHz microwave power to sub-cutaneous targets, while positioned externally adjacent to the targeted tumor. The applicator consists of an ‘S’ shaped radiating element tuned to operate at 2.45 GHz terminating a feeding coaxial transmission line. To direct the radiation toward the tumor, the antenna is coupled with a hemi-cylindrical copper reflector. Cooling water is circulated through the reflector tube, which returns through an outer polyimide sheath, thus providing a closedloop coolant flow circuit. The applicator was designed to be ~70 cm in length to allow for insertion within the vertical high-field scanner. Based on the estimated insertion loss of the coaxial cable (UT47C, Microstock Inc.) at 2.45 GHz, and the cable length of approximately 70 cm, we estimate approximately 72% power applied at the connector of the applicator is transferred to the antenna and radiated into the tissue. Considering 6 ft long RG400 cable between power meter and applicator, it is estimated that approximately 53% of power measured at the power meter is transferred into the tissue. Figure 2 illustrates the applicator geometry and depicts a fabricated antenna.

### *System integration and evaluation in tissue phantoms and* ex vivo *tissue*

The accuracy of MRIT with the 14.1 T high-field scanner at temperatures in the range 40–44 °C was previously found to be on the order of 0.4 °C [31]. Temperatures during ablation can be considerably higher than 50 °C. To validate the transient temperature changes estimated by the PRFS technique during ablative exposures, we performed measurements in tissue-mimicking phantoms and *ex vivo* chicken breast and compared temperature changes from MRIT against fiber optic measurements. Even though the temperature profiles in phantoms and *ex vivo* tissues are expected to be different from heating profiles in tumor *in vivo* (due to the lack of blood flow and heterogeneities), these experiments are informative to assess the system integration and MRIT monitoring accuracy ahead of *in vivo* studies. Transient temperature profiles in phantoms as well as *ex vivo* samples were measured with the use of the proton resonance frequency shift technique during microwave heating. We used a FLASH protocol, with TR = 30.1 ms, TE = 4 ms, FA = 15°, FOV = 30 × 30 mm^2^, slice thickness = 1 mm, three axial slices, and in-plane pixel size 0.2344 × 0.2344 mm^2^.

**Figure 3.**
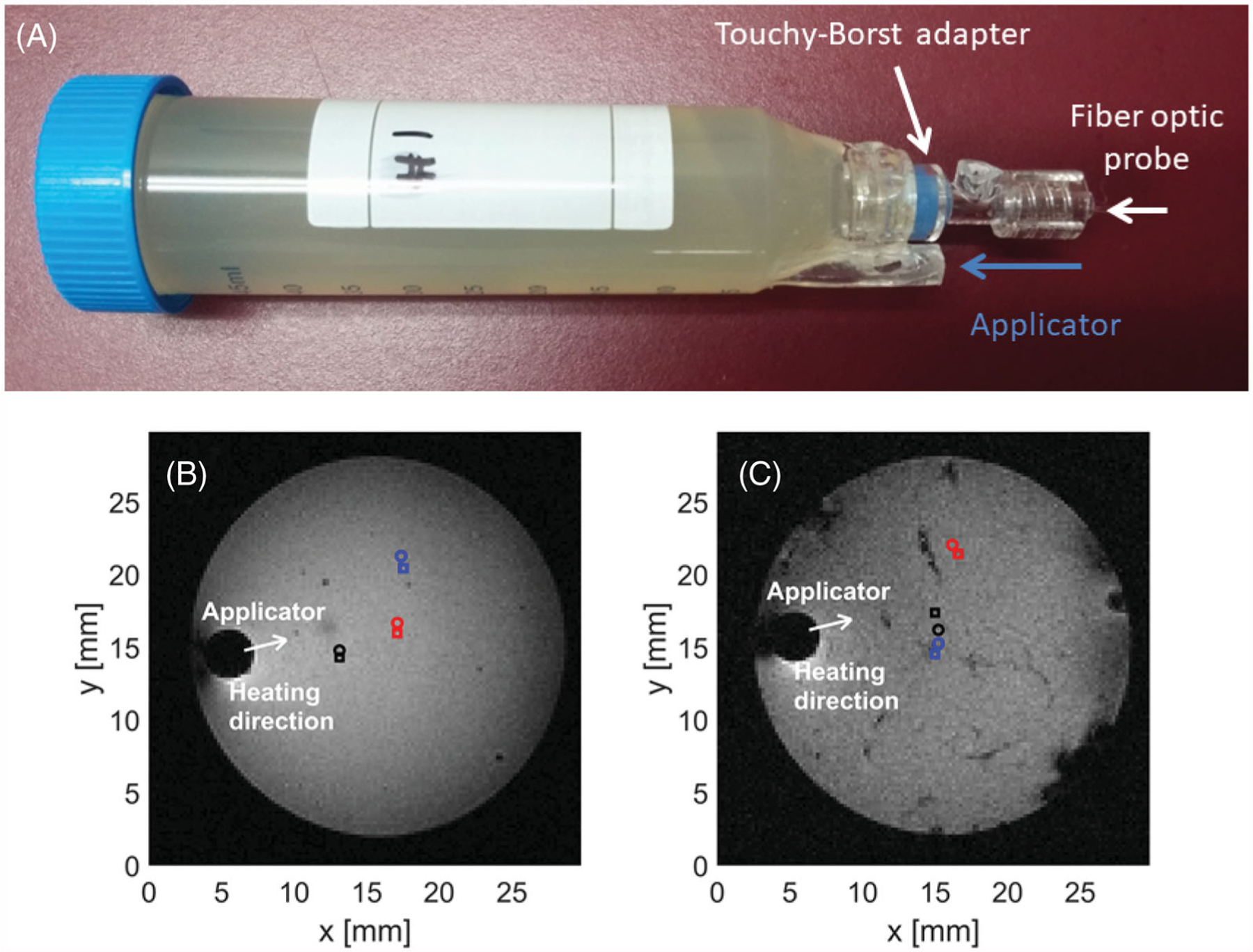
Holding template (A) for phantom or *ex vivo* tissue in MRI scanner. Axial slices of phantom (B) and chicken breast (C) samples loaded in template along with marked positions of applicator and fiber optic sensors.

An agar phantom was prepared according to the formulation previously reported [15,31,32], consisting of 74.75% deionized water, 23% sugar, 0.25% sodium chloride, and 2% agar based on volume. To increase the brightness of the phantom in MRI images, we also added 0.041 g of copper sulfate. Figure 3 depicts a custom template developed for microwave heating experiments in agar phantoms and *ex vivo* chicken breast samples. The sample is loaded from the top (left side in Figure 3(A)) and the microwave applicator and fiber optic probe are introduced from the bottom (right side in Figure 3(A)), with the option of locking a fiber optic temperature sensor in place with a Touhy-Borst adapter. This enables careful registration of the applicator position relative to the fiber optic temperature probes.

Magnitude images in Figure 3(B,C) were acquired with the use of T1-RARE imaging sequence with relaxation time, TR = 1700 ms, echo time, TE = 4.5 ms, number of averages 2, flip angle, FA = 90°, field of view, FOV = 30 × 30 mm^2^, slice thickness 1 mm, and in plane pixel size = 0.11 × 0.11 mm^2^.

### Animals and anesthesia

Experiments were approved by the local institutional animal care and use committee (IACUC) under protocol number 4451. A mouse model of aldosterone producing adenoma was prepared by injecting six million HAC15 cells (human adrenal gland carcinoma cells, ATCC: CRL-3301) subcutaneously in the right hind flank of nude mice (nu/nu, Charles River strain #490). Starting at day seven post-injection, the 2-dimensional tumor size was estimated using calipers. When the tumor size reached 5 mm in one direction, the mice were treated. A total of six nude mice were selected for heating procedures. On the day of experiment, the mice were anesthetized using isoflurane (5% induction, 1–2% maintenance) and maintained under anesthesia for the duration of the treatment.

### Computational modeling of microwave ablation of subcutaneous HAC15 tumors with a directional microwave applicator

**Figure 4.**
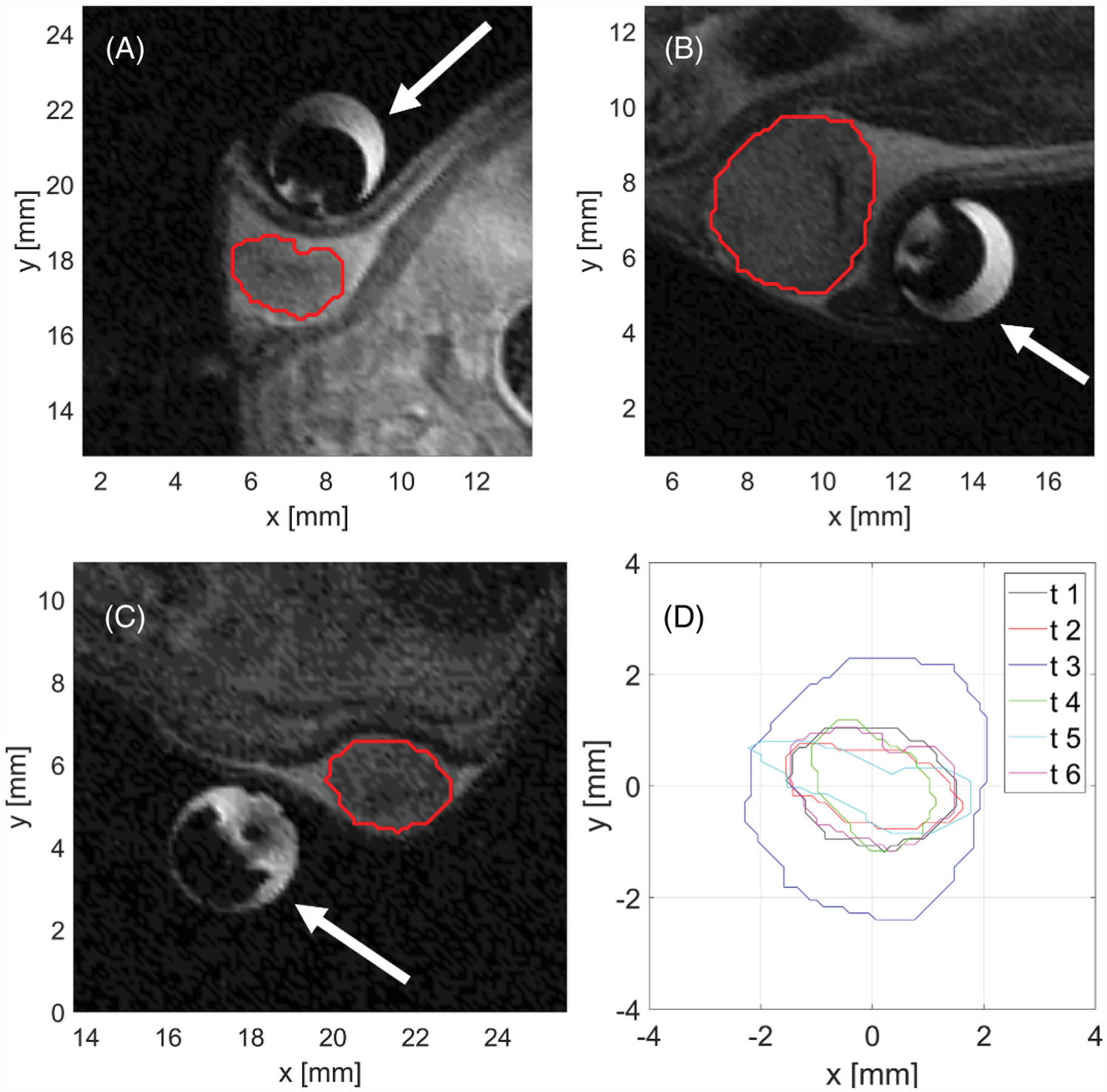
Example (A–C) anatomic MR images of subcutaneously implanted HAC15 tumors (red contour) and the microwave ablation applicator (white arrow). The boundaries of all six treated tumors (D) in a central axial plane through the tumor.

Figure 4 illustrates the anatomy of example HAC 15 tumors in mice, prior to delivering ablation treatment. Tumors ranged in maximal diameter between 2–5 mm.

To evaluate the suitability of the directional microwave applicator for heating of subcutaneous tumors, observed tumor shapes and sizes as illustrated in Figure 4 were compared with the simulated ablation zones estimated by a finite element method (FEM) computational model. The coupled time-harmonic electromagnetic and transient heat transfer problem was iteratively solved in a homogenous block of tissue around applicator. At the outer boundaries of model, the Sommerfeld radiation scattering boundary and thermal insulation boundary conditions were applied. In addition, water-based cooling of the applicator was modeled by convective heat flux boundary condition at the outer surface of applicator with heat-flux coefficient *h* = 200 W/m^2^/K and external temperature 20 °C.

For the time stepping scheme, we used low order (maximum 2) backward differentiating formula (BDF) with relative tolerance within time domain solver was set to 0.005 to aid the convergence of numerical computations. We used a free tetrahedral mesh with the smallest element size of 0.1 mm around the input port to the applicator, gradually increasing the size to 0.25 mm around the applicator shaft and allowing the element size to be gradually increasing up to 3 mm for space more than 1 cm away from the applicator shaft. These parameters were chosen based on the result-convergence method, where the changes in simulated temperature and electric profiles were monitored for simulations with decreasing mesh size and relative tolerance and the parameters resulting in output changes smaller than 1% of initial differences were employed.

The tissue block around the applicator was modeled with dielectric and thermal properties of *ex vivo* liver, due to lack of data on HAC15 tumors in literature, and liver being commonly accepted as a surrogate tissue for performance evaluation of ablation devices. Baseline properties of tissue were taken from IT’IS tissue database [36], temperature dependencies of dielectric properties from our previous study [37], and temperature dependencies of thermal properties from [12]. The ablation zone boundary was estimated by a thermal dose of 240 cumulative equivalent minutes at 43 °C (CEM43). For each of the six treated tumors, the required ablation duration, for which the simulated ablation fully encompassed the tumor was found and the Dice Similarity Coefficient (DSC) was computed the between tumor and simulated ablation. For tumors fully encompassed by predicted ablation zone, DSC allows for relative comparison of collateral damage (i.e., thermal damage to non-target tissues) between tumor cases, where higher DSC stands for higher similarity between ablation and tumor shape and therefore lower collateral damage. Simulations were carried out for 30 W and 50 W of applicator input power.

### In vivo *heating procedure*

After inducing anesthesia, the mice were placed in an acrylic fixture which allows for positioning of the small animal, temperature sensor, breathing mask, and microwave applicator in a defined relationship within MRI probe, which is to be loaded to the 14.1 T MRI scanner. The microwave applicator was then positioned on the skin of the mouse next to the subcutaneous HAC15 tumor and fixed with the tape such that the tumor was in the direction of the applicator’s heating pattern. A rectal thermocouple probe (SA Instruments, Inc.) was used to capture the core temperature of the mouse just before starting the heating procedure to allow absolute temperature measurements *via* MRIT.

**Figure 5.**
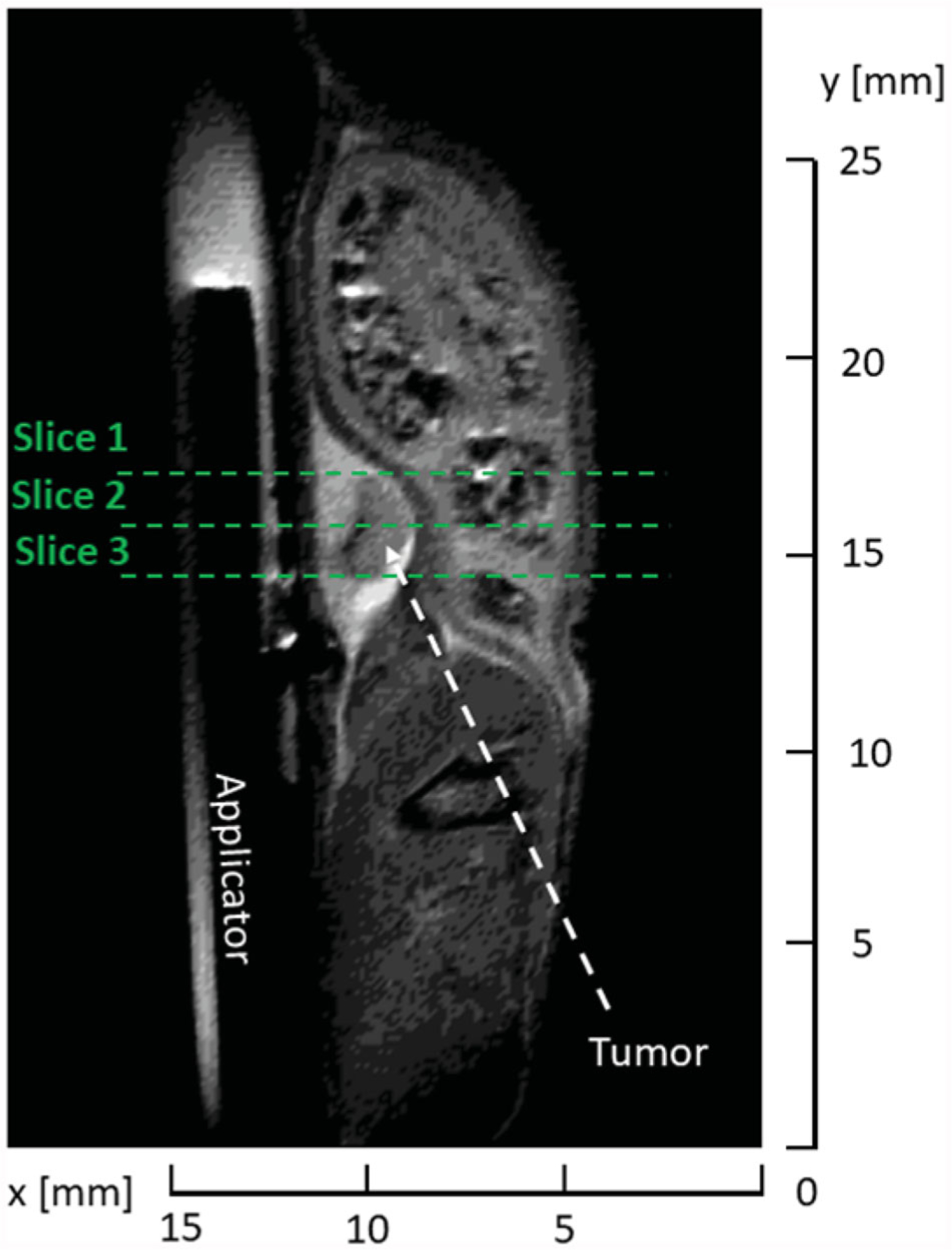
T2-turboRare anatomical image with highlighted positions of axial slices for real-time temperature monitoring.

Once the MRI probe was loaded in the scanner, initial T2-TurboRARE scans were obtained in the axial, sagittal and coronal view to verify the position of the microwave applicator with respect to the tumor as shown in Figure 5 for sagittal plane. Parameters of the T2-TurboRARE imaging sequence were relaxation time, TR = 1500 ms, echo time, TE = 14 ms, number of averages 2, flip angle, FA = 90°, field of view, FOV = 30 × 30 mm, slice thickness = 0.5 mm, and in-plane pixel size = 0.11 × 0.11 mm. Transient temperature profiles were measured with the use of same PRFS method and FLASH protocol as in case of *ex vivo* samples (TR = 30.1 ms, TE = 4 ms, FA = 15°, FOV = 30 × 30 mm^2^, slice thickness = 1 mm, three axial slices, and in-plane pixel size = 0.2344 × 0.2344 mm^2^).

Prior to the initiation of microwave heating, we selected several points along the tumor boundary as regions of interest (ROI), where temperature was monitored in real time. Four points were assigned along the tumor boundary in each of the 3 slices shown in Figure 5. Special care was taken such that the points resided within the tumor volume and no further than 0.5 mm from the tumor boundary. In each plane, one point was placed adjacent to the applicator and three other points were placed equidistantly along the remaining tumor edge. Heating was terminated when the threshold temperature of 55 °C was reached in all ROIs. During heating, 45 W input power, as measured at the power meter, was used, which translates to approximately 23.9 W was radiated into tissue. Following completion of ablation, the imaging coil was withdrawn, animals recovered from anesthesia and returned to housing. The animals were euthanized 24 h following delivery of ablation; tumor samples were excised and fully immersed into solution of triphenyl tetrazolium chloride (TTC) for 1 h to identify any living tumor cells with functioning mitochondria [38]. Following staining, photos of tumor samples were taken for quantitative analysis of tissue surface color change to assess the ablation extent.

## Results

### Comparison of simulated ablations and target tumor shapes

**Figure 6.**
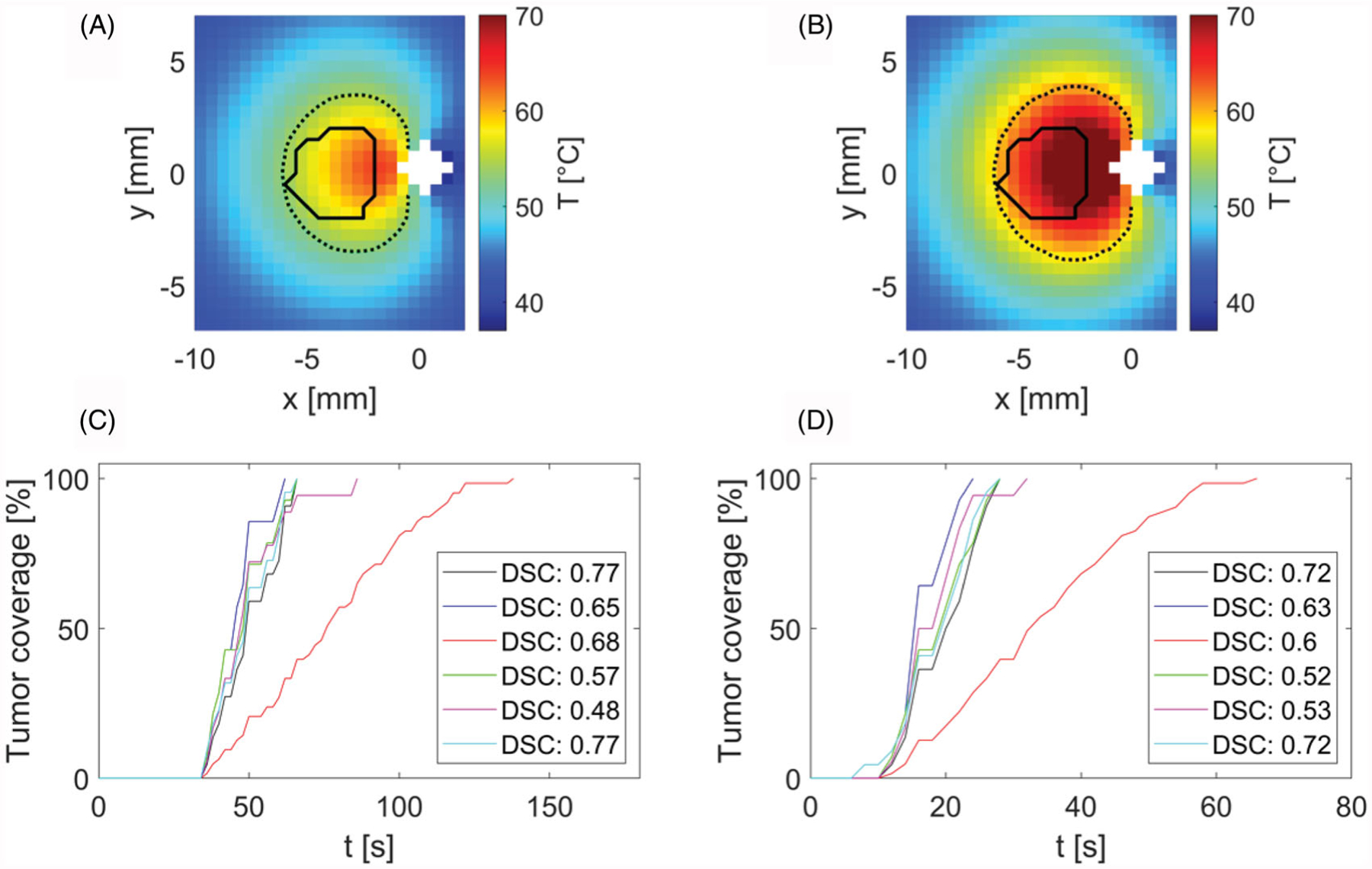
Comparison of simulated ablation zone at 30 W (A,C) and 50 W (B,D) input power (as measured at the power meter) with target tumor boundaries in a central axial plane. While (A,B) illustrate sample temperature distributions and tumor boundaries, (C,D) provide the Dice Similarity Coefficient (DSC) between the simulated extent of the ablation zone and the corresponding tumor boundary, for each of the six cases. White space in (A,B) denotes the applicator. Solid lines in (A,B) denote the tumor boundary and dashed lines represent the CEM43 = 240 min isodose line.

Figure 6 shows the results of the shape comparison between tumors segmented from acquired anatomical images and simulated ablation zones at time when the tumor was first fully encompassed by a zone with thermal dose equal to or exceeding 240 CEM43. Comparisons were performed at power levels of 30 W and 50 W, as measured at the power meter. Figure 6(A,B) illustrate the shape overlap of an example tumor and respective temperature map at the time when tumor got fully encompassed by zone with 240 CEM43 or higher thermal dose. Figure 6(C,D) then illustrate the tumor coverage as a function of time and list similarity (DSC) between shapes of tumors and simulated ablations at times which were required to fully encompass tumors with a minimum of thermal dose of 240 CEM43.

As can be seen in Figure 6, performed simulations predicted full coverage of all tumors with minimum thermal damage of 240 CEM43 within 60–140 s for 30 W and 22–66 s for 50 W input power.

### Ex-vivo *based validation of MRI thermometry*

**Figure 7.**
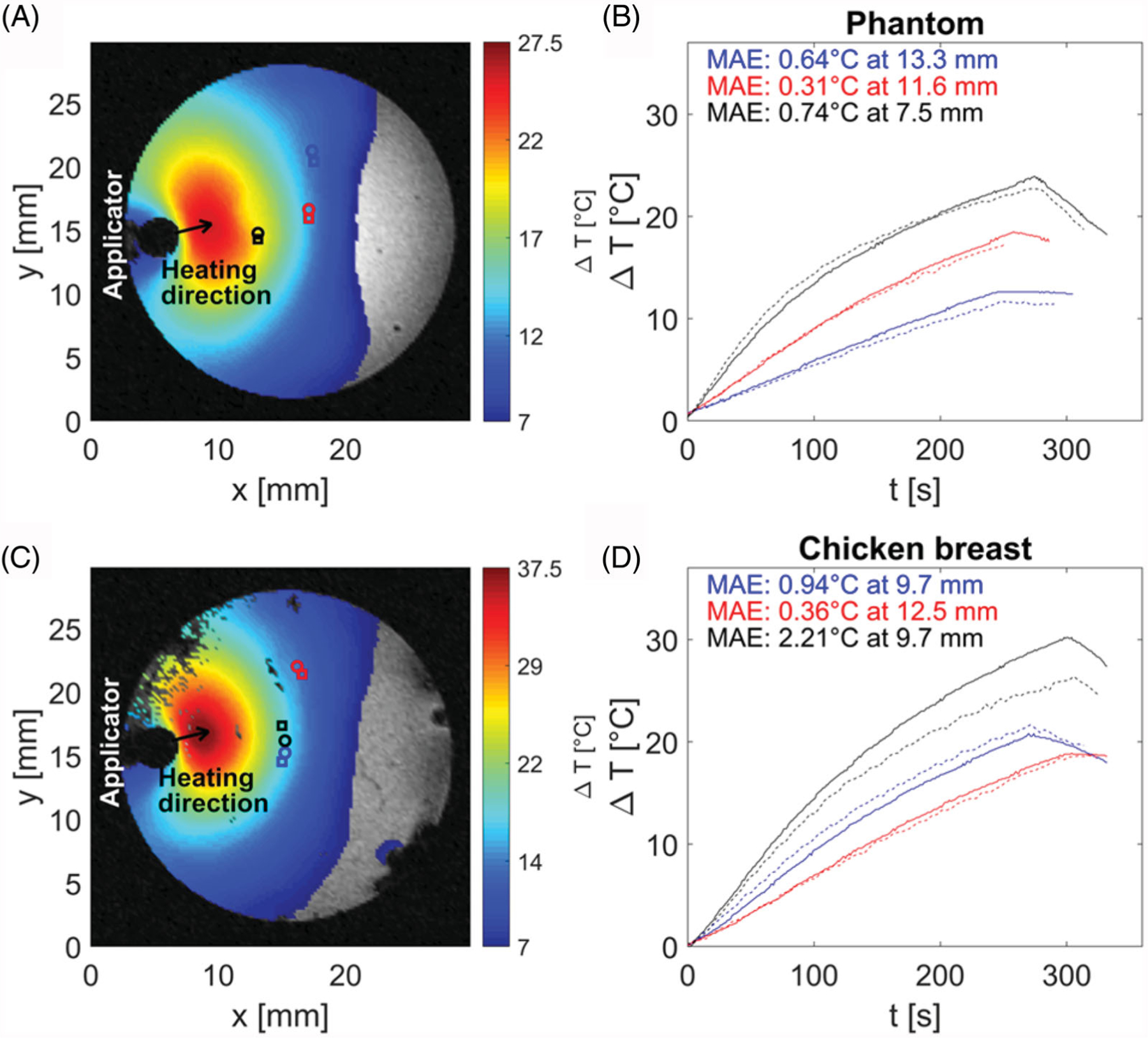
Comparison of temperature profiles during heating in agar phantom (B) and chicken breast (D) along with magnitude images (A,C) with position of applicator, heating direction and positions of fiber optic probes (circle marks) and regions of interest (square marks) for temperature evaluation. Magnitude images also show temperature maps, which were achieved at 240 s after the start of heating. The color of temperature difference curves shown in (B,D) correspond to the color of location markers in magnitude images (A,C).

To validate the MRI thermometry, we performed three heating procedures in agar phantom and another three procedures in *ex vivo* tissue. Heating times were in range of 4–5 min with an average 4.58 min. The update time between subsequent images was 3.86 s based on used sequence parameters. The results of MRIT validation in agar phantom and chicken breast are shown in Figure 7.

During heating phantoms and *ex vivo* tissue, peak temperatures of 67 °C were observed, with average mean absolute error (MAE) between fiber-optic thermometry and MRI of 0.86 °C across all samples. Figures S1 and S2 in supplementary data document illustrate the experimentally measured (with MRI thermometry) and simulated transient growth of thermal profiles.

### In vivo *ablations with MRI thermometry*

**Figure 8.**
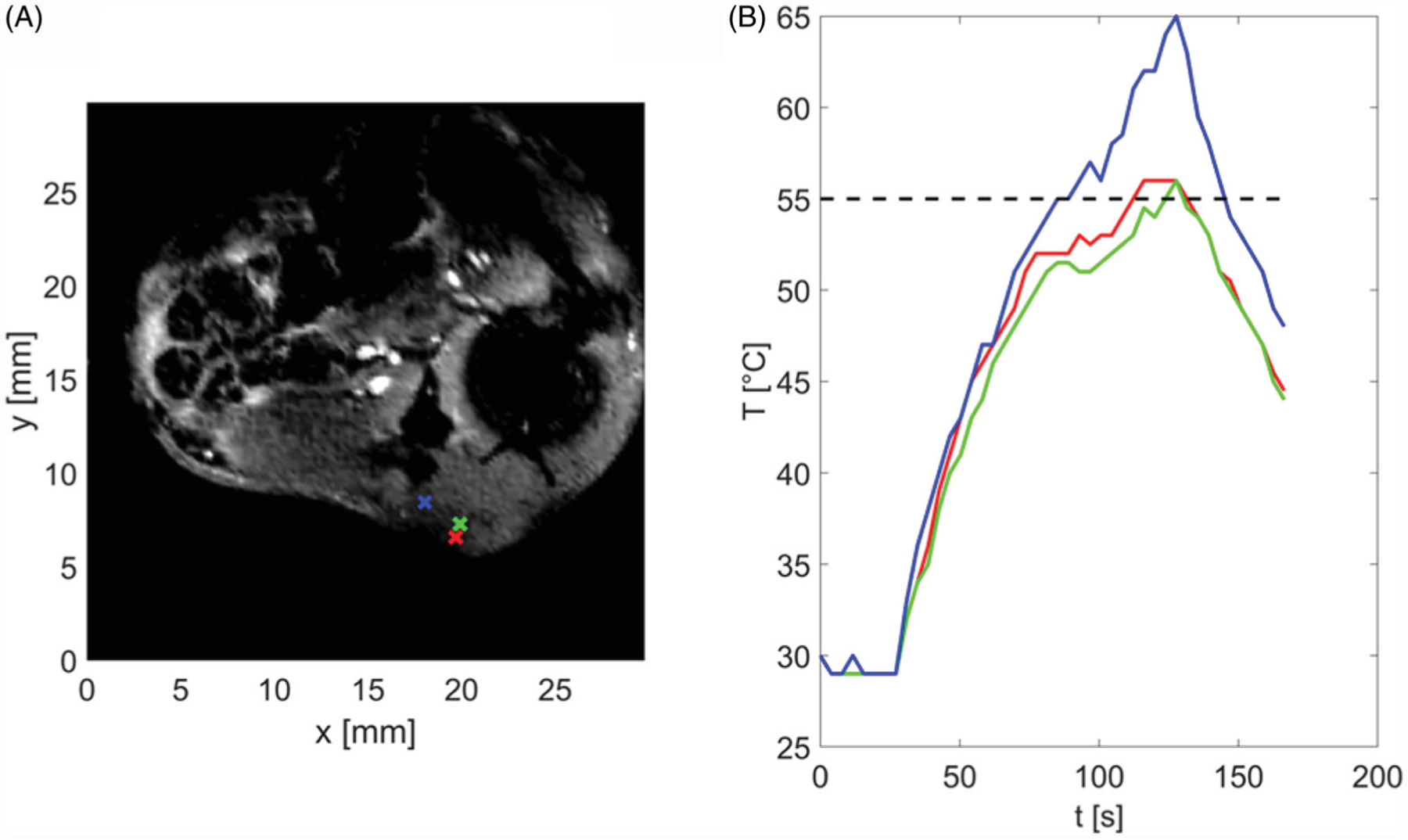
(A) Magnitude image showing the tumor and surrounding anatomy and control points used for temperature monitoring and terminating procedure when temperature reaches threshold of 55 °C. (B) Temperatures estimated at control points shown in subfigure (A). Color of each line corresponds to color of control point in subfigure (A).

Figure 8 shows an example anatomic image of the central slice of tumor in a mouse along with control points at the tumor boundary, where the temperature was monitored in real time and procedure was terminated once temperatures in all positions exceeded a threshold of 55 °C. Due to the tissue varying properties as well as variations in applicator contact with skin across experiments, heating time varied between 103–259 s across experiments (Table 1). The update time between subsequent image samples was 3.86 s, consistent with *ex vivo* experiments.

**Figure 9.**
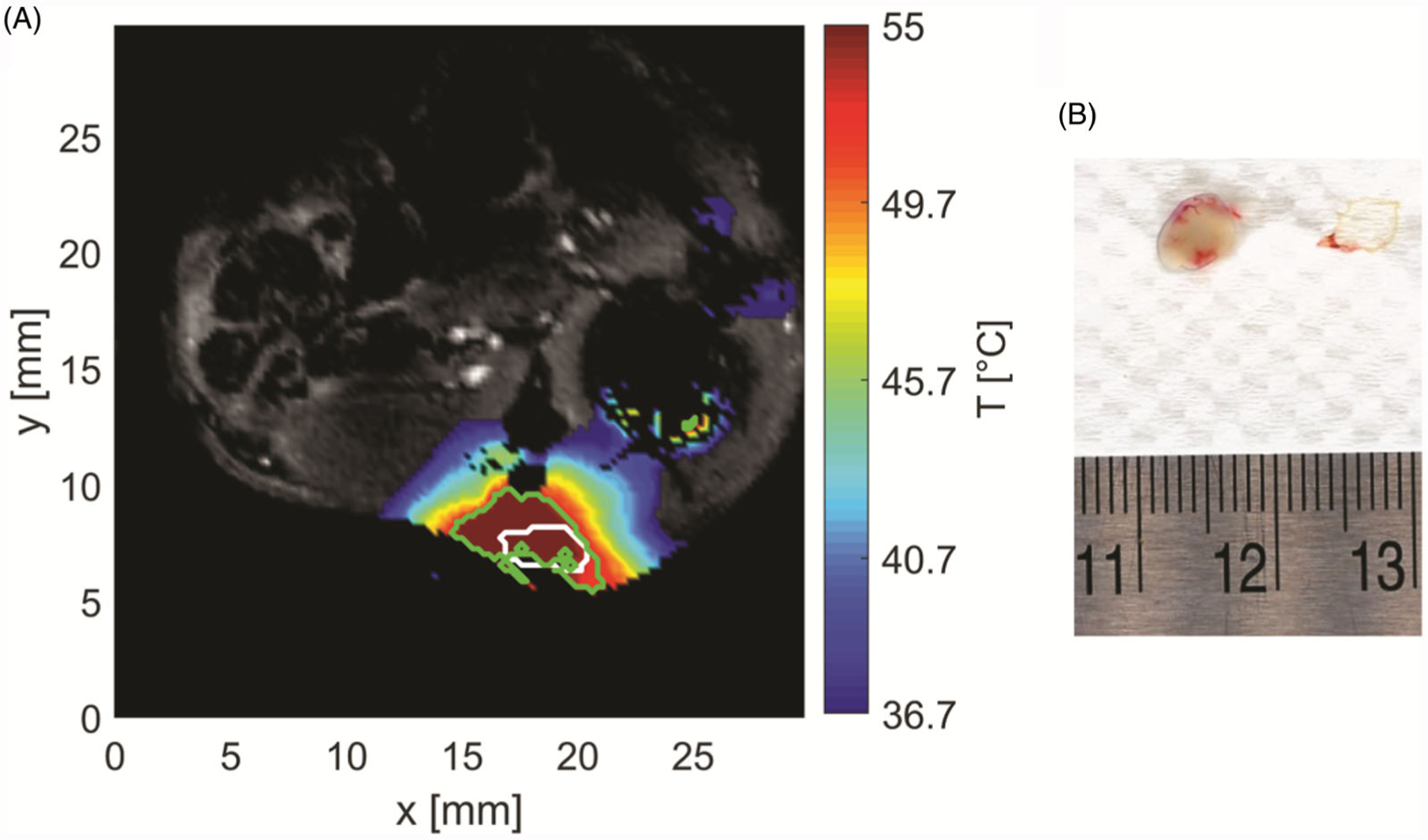
(A) Sample temperature map shown over the magnitude image at the end of ablation. White contour stands for segmented tumor and green contour for area with thermal dose greater than 240 CEM43. (B) Extracted tumor with applied TTC staining (white tissue) and surrounding vessels (red tissue).

Figure 9(A) shows the measured temperature profile in an example tissue slice as estimated by MRIT at 1 min 43 s after the start of ablation, when the applied power was reduced to zero (i.e., when tissue at all control points along the tumor periphery exceeded 55 °C). The white contour represents the segmented tumor boundary and the green contour the boundary of irreversible thermal damage as estimated by 240 CEM43 thermal dose. Figure 9(B) shows the excised tumor with surrounding blood vessels 24 h after ablation and following viability staining with TTC. The pale/white color of tissue is indicative of no viable cells within the treated tumor.

Table 1 provides the volume of tumor in each mouse along with the volume of tumor encompassed by the threshold ablative thermal dose of 240 CEM43, and maximum achieved temperature in tissue as estimated by MRI thermometry.

## Discussion

Simulations predicted that the full coverage of tumors with minimum thermal damage 240 CEM43 is attainable in each tumor case in less than 140 s for 30 W and less than 66 s for 50 W input power. Furthermore, comparison between shape of simulated ablation zone and target subcutaneous tumor shapes from anatomical MRI scans at the time of full tumor ablation yielded average DSC 0.65 ranging from 0.48 to 0.77 for 30 W and average DSC 0.62 with range 0.53–0.72 for 50 W input power. DSC value of 0.7 was previously suggested as an indication of conformity between target shape and thermal contour approximating ablation [39]. The range of our DSC similarities suggests reasonable agreement between tumor and ablation shapes, with some collateral damage to adjacent tissue around tumor.

However, MR images as well as visual assessment after tumor extraction following euthanasia indicated tumors were exclusively surrounded by a layer of fat, which is not a critical structure and can tolerate thermal damage without safety concerns. Moreover, since the target tumor biophysical properties are unknown, simulations were performed in homogenous *ex vivo* liver to assess general ablation shape. The actual heating profiles might be even more favorable for targeted tumor heating in terms of shape similarity, because of possible contrast in dielectric and thermal properties between tumor and fat, where more microwave energy is deposited in volumes with higher dielectric properties (i.e., tumor) and heat is preserved in tumor area due to thermal insulation of surrounding fat layer.

The utilized MRI imaging sequence with 3.86 s update time and average temperature measurement accuracy of 0.6 °C appears to be a suitable monitoring technique for small animal/phantom sample heating. The experiments with phantoms and *ex vivo* chicken breast heating demonstrated the ability of system to heat tissue in regions up to 12 mm from the surface of the applicator to ablative temperatures (i.e., > 55 °C). In one case of chicken breast tissue (black curves in Figure 7(D), the observed MAE was considerably larger (>2x) than in the case of all other measurements. This was due to the rotation of the sample within the sample holder over the course of the experiment.

During 5 out of 6 *in vivo* ablation experiments, temperature at control points exceeded 55 °C following which heating was terminated, with ablation duration ranging between 103–259 s. In 4 out of 6 experiments, more than 90% (range 90.73–97.98%) of the tumor volume (range 5.43–35.92 mm^3^, mean 14.23 mm^3^) was heated to thermal doses exceeding 240 CEM43. In one experiment temperatures recorded over 250 s did not exceed 50.5 °C. In another experiment, MR thermometry data were unusable because of animal movement leading to substantial motion artifacts. In the five experiments, where usable thermometry data was available, thermal dose-based assessment of ablative coverage was in agreement with viability assessment based on TTC staining.

A limitation of the presented system is that it remains susceptible to large errors in temperature estimation due to motion. Presently, motion is managed by appropriately securing the animal within the imaging coil. Future studies may incorporate motion compensation techniques to further improve the robustness of MRI thermometry. We note that the utilized computational model is not a perfect representation of the *in vivo* scenario, for instance the dielectric and thermal properties of this specific tumor type are not known. Nevertheless, the model is instructive for characterizing the approximate thermal profiles in soft tissue and thereby provide complementary information to the temperature profiles acquired during MR thermometry. While we have developed a suitable antenna for ablation of small tumor targets and characterized its performance, it is possible, that other applicator designs with different radiation patterns might also be appropriate for ablation of small tumor targets, and characterization of such devices is warranted ahead of their use in experimental studies.

## Conclusion

We have developed a system for delivering microwave ablation to subcutaneous tumors in small animals integrated with MRI for real-time volumetric thermometry. We verified the accuracy of MRI-derived temperature measurements in agar phantom and *ex vivo* tissue, with an average mean absolute error of 0.86 °C across all samples. The system was further applied for *in vivo* heating of subcutaneously implanted HAC15 tumors in nude mice, where the ability to monitor temperatures at the tumor boundary allowed us to terminate the heating at a time specific to the geometry of each targeted tumor, and therefore to deliver target specific energy.

## Supplementary Material

Supplementary material

## Figures and Tables

**Table 1.** Achieved coverage of tumor by thermal dose of at least 240 CEM43 and maximum estimated temperature in tumor for 5 out of 6 mice.

Mouse	V_tumor_ [mm^3^]	V_cov_ [mm^3^]	V_cov_/V_tumor_ [%]	T_max_ [°C]	Heating time [s]
1	9.06	0	0	50.5	259
2	12.46	11.81	94.71	70	103
3	35.92	22.02	61.31	66.5	150
4	8.29	7.52	90.73	59	134
5	5.43	5.32	97.98	67.5	135

In 1 mouse, thermometry data were unusable because of excessive sample movement.
