# Supplementary material for: System for delivering microwave ablation to subcutaneous tumors in small-animals under high-field MRI thermometry guidance
Source: Int J Hyperthermia. Author manuscript; Available in PMC 2022 Dec 2. (PMC9717487; doi:10.1080/02656736.2022.2061727)
Supplement: Supplementary material [file NIHMS1818679-supplement-Supplementary_material.docx]

**System for delivering microwave ablation to subcutaneous tumors in small-animals under high-field MRI thermometry guidance – Supplementary data**

*Running Title: Small-animal MRI-integrated microwave ablation*

Jan Sebek^1,2^, Tej B. Shrestha^3^, Matthew T. Basel^3^, Faraz Chamani^1^, Nooshin Zeinali^1^, Ivina Mali^4^, Macy Payne^4^, Sarah A. Timmerman^3^, Pegah Faridi^1^, Marla Pyle^3^, Martin O’Halloran^5^, M. Conall Dennedy^5^, Stefan H. Bossmann^6^, Punit Prakash^1*^

1) *Department of Electrical and Computer Engineering, Kansas State University*

*Manhattan, KS 66506, USA.*

2) *Department of Circuit Theory, Czech Technical University in Prague, Prague,*

*Czech Republic.*

3) *Department of Anatomy and Physiology, Kansas State University, Manhattan, KS 66506, USA.*

4) *Department of Chemistry, Kansas State University, Manhattan, KS 66506, USA.*

*5) College of Medicine, Nursing and Health Sciences, National University of Ireland Galway, Rep. of Ireland*

*6) Department of Cancer Biology, University of Kansas Medical Center, Kansas City, KS 66160 USA.*

**Author to whom correspondence should be addressed:* Punit Prakash, 3078 Engineering Hall, 1701D Platt St, Manhattan, KS 66506, USA. Email: [prakashp@ksu.edu](mailto:prakashp@ksu.edu)

**Illustration of spatial heating profile of utilized directional MWA applicator**

Spatial heating pattern of designed and fabricated directional MWA applicator is illustrated in Figure S1 by actual temperature measurements in 3 axial planes during heating of *ex vivo* chicken breast sample for the MRIT validation purposes.

| *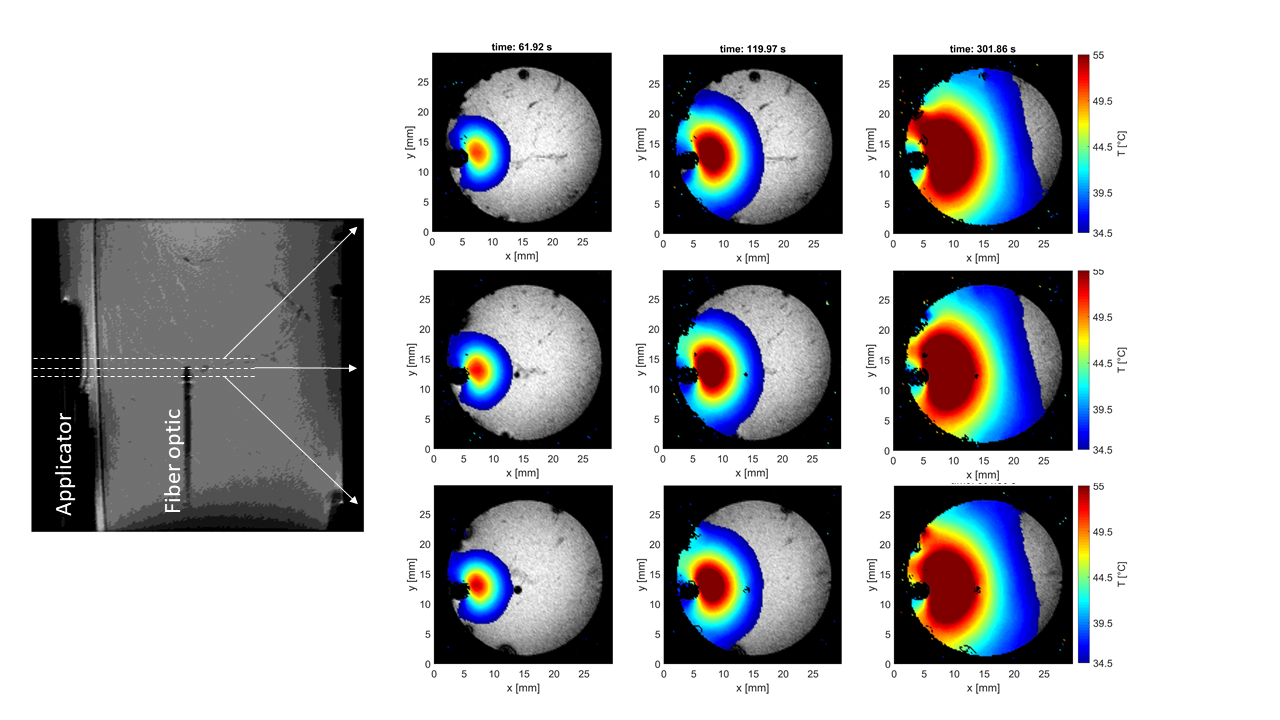* |
| --- |
| **Fig. S1** Illustration of heating profile in 3 axial planes (shown in left figure) from MRI thermometry during *ex vivo* chicken breast heating at 1 min, 2 min and 5 min.. |

Full shape of heating pattern is illustrated in Figure S2 as estimated with use of computational model of applicator in *ex vivo* liver as described in main paper.

| *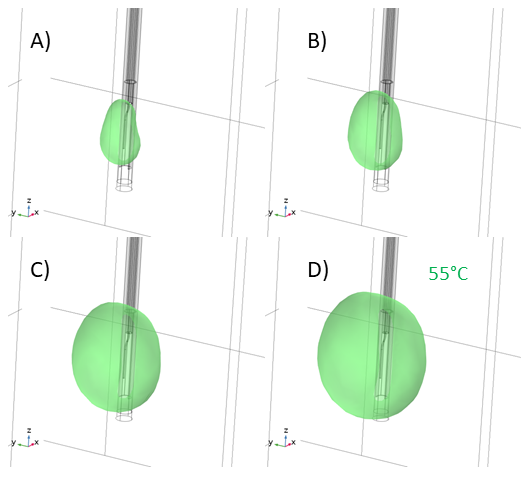* |
| --- |
| **Fig. S2**  Temperature iso-surface for 55°C at A) 30s, B) 1 min, C) 3 min, and D) 5 minutes. |
